# CMC Strategies and Advanced Technologies for Vaccine Development to Boost Acceleration and Pandemic Preparedness

**DOI:** 10.3390/vaccines11071153

**Published:** 2023-06-26

**Authors:** Maria Monica Castellanos, Hervé Gressard, Xiangming Li, Claudia Magagnoli, Alessio Moriconi, Daniela Stranges, Laurent Strodiot, Monica Tello Soto, Magdalena Zwierzyna, Cristiana Campa

**Affiliations:** 1 Drug Product Development, Vaccines Technical R&D, GSK, 14200 Shady Grove Road, Rockville, MD 20850, USA; mmcastellanosm@gmail.com; 2 Project & Digital Sciences, Vaccines Technical R&D, GSK, Rue de l’Institut 89, 1330 Rixensart, Belgium; herve.n.gressard@gsk.com; 3 Drug Substance Development, Vaccines Technical R&D, GSK, 14200 Shady Grove Road, Rockville, MD 20850, USA; xiangming.x.li@gsk.com; 4 Analytical Research & Development, Vaccines Technical R&D, GSK, Via Fiorentina 1, 53100 Siena, Italy; claudia.x.magagnoli@gsk.com; 5 Drug Product Development, Vaccines Technical R&D, GSK, Via Fiorentina 1, 53100 Siena, Italy; alessio.x.moriconi@gsk.com (A.M.); daniela.x.stranges@gsk.com (D.S.); 6 Drug Product Development, Vaccines Technical R&D, GSK, Rue de l’Institut 89, 1330 Rixensart, Belgium; laurent.x.strodiot@gsk.com; 7 Drug Substance Development, Vaccines Technical R&D, GSK, Rue de l’Institut 89, 1330 Rixensart, Belgium; monica.x.tellosoto@gsk.com; 8 Project & Digital Sciences, Vaccines Technical R&D, GSK, Via Fiorentina 1, 53100 Siena, Italy; magdalena.a.zwierzyna@gsk.com; 9 Vaccines Global Technical R&D, GSK, Via Fiorentina 1, 53100 Siena, Italy

**Keywords:** CMC acceleration, pandemic preparedness, vaccine platform, modeling and digital tools

## Abstract

This review reports on an overview of key enablers of acceleration/pandemic and preparedness, covering CMC strategies as well as technical innovations in vaccine development. Considerations are shared on implementation hurdles and opportunities to drive sustained acceleration for vaccine development and considers learnings from the COVID pandemic and direct experience in addressing unmet medical needs. These reflections focus on (i) the importance of a cross-disciplinary framework of technical expectations ranging from target antigen identification to launch and life-cycle management; (ii) the use of prior platform knowledge across similar or products/vaccine types; (iii) the implementation of innovation and digital tools for fast development and innovative control strategies.

## 1. Introduction

It is well known that the COVID-19 vaccination has substantially impacted the course of the pandemic, saving tens of millions of lives globally [1]. First COVID vaccines were developed and made available in 350 days [2], an unprecedented velocity. The conventional product development approaches and supply could not deliver vaccines in the timeframe aligned with pandemic needs; therefore, parallel accelerated clinical and CMC (chemistry, manufacturing, and controls) development efforts were executed, with investment in manufacturing/supply at risk, even before the demonstration of the safety and efficacy of the vaccines. Pandemics need rapid vaccine development and a large, sustainable supply chain to meet global demand, leading to initiatives such as COVAX [3] and unprecedented collaborations across different companies (e.g., joining efforts in trade associations for the development and supply of specific vaccines). The global demand was an extraordinary challenge with respect to other outbreaks (e.g., Ebola, MERS, meningitis, etc.), which impacted a relatively small population.

The worldwide impact of COVID has also evidenced the need to have harmonized positions and implementation pathways regarding CMC acceleration across different regulators and manufacturers. This has resulted in several initiatives, including COVAX workshops [4] as well as discussions and positions by ICMRA [5] and WHO [6], all supported by trade associations such as VE, IFPMA and EFPIA [7,8,9]. Interestingly, well before the COVID-19 emergency, regulators and industry had already established a dialogue on CMC acceleration principles to rapidly address unmet medical needs without compromising the safety and efficacy of products. This resulted in publications highlighting risk-based approaches that could be successfully applied to the COVID pandemic [10,11].

Nevertheless, significant challenges remain in the pre- and post-approval regulatory framework and expectations across different regions, which represent an issue for future pandemics. The challenges and learnings associated with vaccine development and life cycle were published in a series of articles with some recommendations that cover CMC technical, compliance, and regulatory aspects ([12] and references above).

In addition, CEPI has articulated an aspirational goal that vaccines should be ready for initial authorization and manufacturing at scale within 100 days of recognition of a pandemic pathogen; this further shortens development timelines compared to COVID vaccines [2] to contain the spread of the pathogen and limit the human and socio-economic impact as much as possible.

This review will provide an overview of critical acceleration enablers, focusing on vaccine CMC development and grounded on published information as well as on experience with multiple vaccine platforms and technologies. The presented strategies and tools are considered beneficial not only for preparedness for the next pandemic but also for the sustainable acceleration of vaccines in development.

## 2. Key Enablers of Acceleration and Pandemic Preparedness

**Figure 1:**
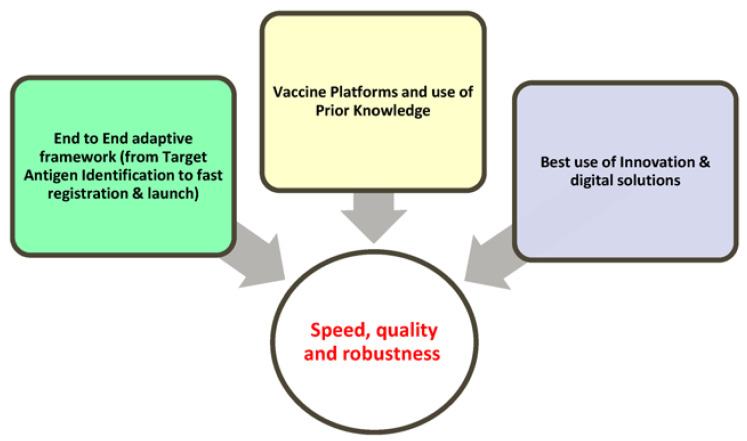
Strategic pillars for pandemic preparedness and acceleration.

After an extensive assessment of the literature and cross-company position papers, it can be concluded that rapid development without compromising safety and efficacy requires the synergic implementation of three strategic pillars, as reported in Figure 1:

1. A holistic, cross-disciplinary framework with clear expectations and actions for each relevant area, from target antigen identification to fast registration and launch.
2. The establishment of vaccine platforms, for DS (drug substance), DP (drug product), and analytics that are ready for immediate use in both development and GMP areas. In this context, it is critical to build and use prior knowledge to mitigate risks and facilitate the decisional process.
3. The best use of innovation, data science, digital solutions, and automation for fast development and innovative control strategies.

**Figure 2:**
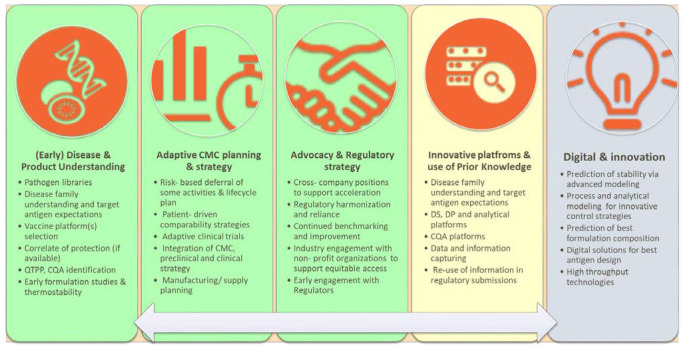
Enablers associated with pandemic preparedness and sustained acceleration (green color: related to end-to-end adaptive framework; yellow color: related to prior and platform knowledge; grey color: related to digital and innovation).

Figure 2 provides a graphical summary of the enablers deemed relevant for pandemic preparedness and sustained acceleration for each of the three areas highlighted in Figure 1 as well as showing the importance of interconnection and fast governance.

In the subsequent sections, some considerations on CMC holistic frameworks will be provided, but most of the content will be on prior and platform knowledge as well as on digital strategies and innovation; according to the literature analysis, these two areas require more extensive review and critical assessment. For the same reason, it will not be possible to cover all the individual elements reported in Figure 2 in a single review; therefore, the focus will be given to highly impactful factors requiring reflection.

### 2.1. Holistic Framework of Expectations from Candidate Identification to Launch

#### 2.1.1. Early Disease and Product Understanding

Compared to biotherapeutics, there are several specific features of vaccines that represent challenges to the design and development of a vaccine in the context of a pandemic emergency. First, for a given pathogen, several vaccine types can be considered (e.g., to address the COVID emergency, mRNA, sub-unit proteins, viral vectors, and inactivated viruses were used for the same viral target). This implies the re-use of prior knowledge considering the selected vaccine type. Second, formulation compositions may be vaccine-type dependent (e.g., the presence of adjuvants in protein-based vaccines) and may be multi-component due to the nature of the disease. Finally, correlates of protection may not be known during early development, leading to initial assumptions related to the relevance of non-clinical models.

However, the development of a vaccine based on Quality by Design (QbD) principles [13] and prior knowledge [10,11] can be carried out in a mindful and efficient manner. Contributing factors include the following:

- The screening of a variety of vaccine types, even for the same disease, offers the flexibility of selecting the most efficient and developable platform based on prior knowledge and actual preclinical results. The extent of prior knowledge that can be re-utilized for different vaccines is dependent on the vaccine type. For instance, mRNA and viral vectors typically allow an extensive use of product, analytical, and process platforms, while subunit proteins require more product-specific customization, depending on the cell line selected, specific protein features, and consequent purification, formulation, and analytical panel.
- Prior knowledge of toxicological profiles and of overall safety-related quality attributes, if available, may be used to shorten vaccine development timing during the pandemic while potential critical quality attributes (pCQAs) for efficacy may require specific studies.
- In early phases, in parallel with clinical confirmation, it is, therefore, appropriate to secure an understanding of product characteristics that are relevant for efficacy. In addition, clinical studies may be designed to support evolving product knowledge in accelerated scenarios and future changes in the life cycle when reliance on nonclinical models is not possible and when information from similar products cannot be used. Those studies include dose-ranging for appropriate dose selection (i.e., the ideal dose should be higher than the minimum demonstrated active dose [10,13]) and tailored studies where relevant structural changes are interrogated.
- The extensive knowledge about CQAs and appropriate analytical strategy and process design for monitoring and controlling those CQAs enables comparability studies to be conducted. If desired, this enables the ability to carry out various steps in vaccine development in parallel or to support the introduction of new manufacturing sites to grant global supply.
- The use of modeling strategies and artificial intelligence tools helps in streamlining and rationalizing experimental activities to be performed. Applications relevant to pandemic preparedness include, for instance, formulation and antigen design optimization, stability modeling to support rapid shelf-life definition, and advanced control strategies to accelerate process understanding and commercial manufacturing readiness. More elements of digital strategies will be reported in Section 3.

The QbD framework built throughout the CMC development is a key acceleration enabler as it allows an understanding of what needs to be prioritized and why, identifying priorities based on patient needs with risk-based life cycle plans; this structured information capturing and planning enriches the knowledge base so that data across vaccines can be more easily reused [13]. A centralized and digitalized knowledge base is particularly useful in the context of a vaccine platform. The product experts will pay particular attention to the product-specific areas while leveraging the platform knowledge for cross-product aspects and tackling virus variants. Once the vaccine development is completed, the QbD knowledge materialized through the control strategy can be transferred to manufacturing. In life-cycle management (LCM), new information can be added, and new vaccines could profit from prior knowledge built.

#### 2.1.2. Adaptive CMC Planning and Technical Advocacy Learnings

Three years after the COVID-19 pandemic onset, and based on the experience gained, vaccine manufacturers and health authorities have the strategic imperative to accelerate CMC cycle times to bring vaccines to populations faster, in order to tackle unmet needs and be ready for the next pandemic [2,5,7,8,9].

To achieve this ambition, coordinated dialogue across different manufacturers is key to generating a common and objective set of proposals and points of attention to be shared with regulatory agencies in different world areas. Several proposals have been published by industry groups to address technical and regulatory challenges, including risk-based process validation, comparability, and stability strategies, post-approval changes, national control laboratories testing, and regulatory harmonization [7,8,9,14]. The tackling of new variants, which is still a potential concern for COVID-19, deserves particular attention. Key enablers for readiness to address virus mutations include advanced approaches for antigen design (see Section 2.3.1) and use of prior and platform knowledge (see Section 2.2) as well as the establishment of fast-track globally converged regulatory mechanisms (e.g., based on reliance and mutual recognition) [8,9].

Of note, COVID learnings from regulatory agencies have also been published [15], highlighting the opportunity for the early and continuous engagement of vaccine developers with the regulators, the importance of increased international regulatory cooperation for CMC/GMP during the pandemic, and the importance of early investment in GMP for production and testing sites for vaccines. In the cited paper [15], it is clearly stated that “CMC requirements are not waived to accelerate EU COVID-19 vaccine approvals. The pandemic requires an alternative, flexible approach for data provision in the context of the benefit/risk judgement.” In this context, the relevance of good product understanding is considered “a prerequisite to a flexible process validation approach as product quality can then be reliably monitored as part of routine batch release specifications”, in alignment with the reflection reported in the previous paragraph and with cross-company discussions.

From an individual company perspective, to achieve pandemic preparedness, it is critical to ensure continued prioritization of the investments in acceleration initiatives on relevant vaccine platforms, incremental investment in new technologies/resources to drive further acceleration potential, and a commitment to test technologies/platforms across multiple programs.

The next two sections will focus on platform knowledge use and on new technologies to accelerate vaccine access, providing a view of the published information as well as the authors’ perspectives.

### 2.2. Vaccine Platforms and Use of Prior Knowledge

Prior knowledge is key for accelerated vaccine development and is intrinsically related to platform development. The importance of prior knowledge has been previously discussed in workshops [11] and recognized as a key recommended approach for the development of medicines in a pandemic situation.

Prior knowledge is defined as “an established tool that is explicitly or implicitly used for informing decisions during pharmaceutical development and life-cycle management” [11].

Practical examples of prior knowledge built on vaccine platforms will be discussed in this section. As mentioned above, for a given pathogen, several vaccine modalities can be considered for development. Among those, mRNA and subunit Chinese hamster ovary cells (CHO) platforms have been considered in this paper due to the following reasons:

- They are both suitable for viral targets, which are associated with pandemic threats.
- mRNA provides a fast response to health emergencies, thanks to the relatively simple process and the possibility to extensively reuse product, analytical, and process knowledge across different targets. On the other hand, thermostability and duration of the immune response are still under assessment.
- CHO is a well-established expression system for subunit vaccines, with progressing advances in throughput and increasing use of platform knowledge, also leveraging on mAbs experience. Despite being intrinsically less rapid than mRNA, subunit vaccines represent a key player in the pandemic response due to the robust and scalable product and process development, limited thermostability issues, possibility to support immune response by appropriate adjuvants, and broad industry and patient experience across different diseases.

In the subsequent paragraphs, drug substance, drug product, and analytical considerations will be reported. It is worth noticing that, in addition to individual discipline considerations, the integration and subsequent synchronization of activities are needed to enable the fast reaction to urgent unmet medical needs. This can be achieved through the implementation of a CMC development strategy and expectations grounded on QbD, as described above.

#### 2.2.1. Drug Substance

Establishing a platform is a significant investment in time and resources from the end-to-end CMC teams, an effort that pays off by significantly streamlining new vaccine candidates from discovery to clinic and to market, reducing timelines and limiting risks without impacting qualify and safety. Continuous improvement and evolution of the platform is a must, to keep up with increased pressure to accelerate patient access to pandemic and non-pandemic vaccines. This can be carried out by the re-injection of knowledge acquired with every new product run and the readiness to implement the latest regulatory trends, industry innovations, and technologies.

In the most recent pandemic situation, the mRNA platform has demonstrated its advantage as a rapid vaccine platform with acceptable safety and efficacy [2]. Considering several studies have been available regarding the pandemic-preparedness role of the mRNA drug-substance platform, in this section, we will give a brief summary of mRNA elements while providing more detailed insights focusing on the novel acceleration levers for the CHO recombinant protein platform.

The advantage of the mRNA platform has been attributed to its intrinsic nature of having a fully platformized drug substance process: in brief, the mRNA drug substance production is a cell-free process that starts with the production of plasmid DNA (pDNA), which encodes the target vaccine antigen, followed by mixing with enzymes and nucleotides to allow for its in-vitro transcription into properly capped mRNA. The mRNA is subsequently purified through a variety of chromatography techniques, such as affinity, size-exclusion chromatography, and HPLC-based methods to remove the process-related (e.g., enzymes) and product-related impurities (e.g., double-stranded RNA). The key levers for mRNA drug manufacture related to the further shortening of the development timeline as well as the enhancement of the safety profile can be summarized below: (1) shortening the lead time for pDNA manufacture by replacing fermentation-based process with a synthetic approach; (2) minimizing the presence of reactogenic by-products, i.e., double-stranded RNA, through the optimization of upstream in vitro transcription, such as in vitro enzyme evolution of T7 polymerase [16] and altering nucleoside triphosphate (NTP) ratios during transcription [17], coupled with the inclusion of additional downstream chromatography steps such as reverse phase HPLC and cellulose [18,19].

In the meantime, the recombinant protein platform, especially the CHO expression platform, a well-established platform that demonstrated superior safety and efficacy, has also played a pivotal role in fighting the COVID-19 pandemic, as evidenced by multiple monoclonal antibodies (mAb)—based biologicals rapidly developed at unprecedented speed in clinic and market approval [20,21,22]. As discussed by multiple industrial players in the mAb-based therapeutic area [23,24], this swift CMC development timeline has been attributed to the following three acceleration levers: (i) leveraging prior knowledge, development approaches, and infrastructure; (ii) smart business-risk approaches; (iii) novel cell line development workflows and expression technologies. Similar to mAb-based therapeutics, the recombinant protein (CHO) adjuvant vaccine platform could potentially benefit from the three levers with some special considerations for certain aspects. The next paragraph will focus on prior knowledge and smart risk-taking, while considerations on innovations in cell line development will be captured in Section 2.3.

Compared with the mRNA platform, which is considered an end-to-end platform for vaccine production, the CHO platform is a semi-platform that requires customization of certain steps to the individual vaccine target, especially when it comes to the downstream purification (definition of chromatography resins, buffers, and steps). However, there are some common elements that could be leveraged for further acceleration opportunities. A few examples are listed here: (1) leverage platform and pre-verified/QA-approved raw materials for cell line development and scaling up; (2) templatize the upstream process and generic purification steps (e.g., the method for clarification, viral inactivation, and filtration); (3) predefine high throughput resin screening workflows to accelerate chromatography resin selection; (4) harness prior knowledge for defining critical process parameters and technical risk assessments; (5) utilize pre-existing unit operations and manufacturing sites; (6) platformize the approach to small-scale model qualification, process characterization, and technology transfer between development, GMP, and commercial manufacturing teams; and (7) define template documents to consolidate technical evidence in the right format for fast turnaround into regulatory documents for the different submission stages.

In addition to the use of platform knowledge, further shrinkage of the timeline could be achieved through the willingness of taking smart business risks to change CMC development from a linear workflow to a paralleled and fit-for-purpose way of working [23]. A few examples are listed here: (1) early engagement of the discovery team to initiate CMC development (e.g., cell line development for multiple potential vaccine targets); (2) leverage of intermediate cell substrate (e.g., pool instead of clones) for early non-clinical and clinical drug substance supply; (3) initiation of master cell bank (MCB) manufacture for multiple clones prior to the lead clone nomination; (4) conditional release of MCB with a lean testing panel for Phase I GMP production; (5) implementation of phase-appropriate viral clearance strategy; and (6) production of clinical batches directly at a final commercial facility.

#### 2.2.2. Drug Product

The rapid emergence of new infectious diseases that threaten public health underlines the importance of rapidly moving from the identification of new vaccine candidates from the bench to commercial products. Pharmaceutical formulation science plays a critical role throughout the development, manufacturing, distribution, and vaccination phases. The key objective in the formulation and process development is to deliver a stable, robust, and scalable drug product that conforms to quality and manufacturing requirements ensuring product safety and efficacy [25].

These requirements should be defined by their quality target product profile (QTPP). Over the last two decades, a significant shift has been observed in pharmaceutical quality regulation led by regulators through a series of QbD regulatory initiatives [26]. QbD can also be applied to formulation and process development, providing a great opportunity for understanding and improving product development [27].

The formulation development path includes (1) preclinical studies that include the development of in vitro biological assays and relevant in vivo animal models to assess vaccine antigenicity and immunogenicity; (2) the physical and chemical characterization of the candidate; (3) the development of stability-indicating assays; and (4) the design and optimization of a formulation to maximize the shelf life of the product.

Particularly, mRNA-based vaccines have emerged as a novel modality to develop products at an accelerated pace, a key benefit during a pandemic situation [28]. The formulation of mRNA-based vaccines consists of encapsulating the mRNA drug substance in lipid nanoparticles by combining RNA at low pH with lipids in a mixing device, which typically consists of a microfluidic device or an impingement jet mixer [29]. The formation of lipid nanoparticles (LNPs) occurs by complexation between the negatively charged mRNA with the positively charged ionizable lipid while being stabilized by the helper lipids (cholesterol, phospholipid, and PEGylated lipid). LNPs are subsequently buffer exchanged and filtered, a cryoprotectant is added, and the product is diluted to a target concentration and filled before frozen, typically at temperatures below −60 °C. The LNP technology can be easily adapted to platform approaches, in which the same drug-product process and formulation can be used for most mRNA constructs or targets of interest, particularly when many elements of the mRNA sequence and size are conserved.

In the case of the COVID pandemic, the ability to leverage previous data and technology for different targets enabled the rapid development of vaccines, such as the work on influenza mRNA vaccines and the development of lipid nanoparticles [30,31,32]. Nowadays, many organizations are targeting even shorter development timelines for future vaccines than those achieved with the COVID vaccine [2]. Challenges remain to develop mRNA LNP vaccines that can effectively address a pandemic: developing mRNA LNP vaccines that do not require ultra-low temperatures or frozen storage [33,34], expanding manufacturing capabilities or increasing its throughput [35], and creating strategies to leverage platform data that are widely accepted by regulators or can be rapidly validated [36].

In the case of protein-based vaccines, different formulation considerations and strategies need to be implemented to accelerate their development in a pandemic situation. For instance, the need for an adjuvant in the vaccine composition (such as aluminum salt, and ASO1E, among others) to supplement the activation of the immune system and increase or extend the immune response could represent a challenge for the formulation design [37]. Additionally, due to instabilities in liquid form, many proteins require other approaches for long-term storage, such as lyophilization (freeze-drying). Lyophilization has advantages over liquid formulations in terms of stability, without compromising storage conditions, and logistics for clinical development and marketing. Freeze-dried vaccines must go through three key steps (freezing, primary drying, and secondary drying) under different kinds of stresses. Typically, stabilizing excipients need to be added to the vaccine formulation to protect the protein structure, such as amino acids, salts, proteins, or polymers [38,39]. Finally, lyophilized vaccines must be reconstituted with a solvent or adjuvant before they can be administered to patients, which requires dosing, compatibility, and in-use stability studies. Interestingly, some promising results were published to increase vaccine thermostability by co-lyophilizing a malaria vaccine with AS01 adjuvant [40]. With the recent advances in molecular modeling and digital solutions, it is also possible to use in silico models to predict formulation stability by evaluating the interactions between antigens with different buffers (see Section 2.3 for more detail). Finally, in order to expedite the development of protein-based vaccines, high-throughput screening methods and standard protocols to interrogate specific products and adjuvant CQAs have been developed.

Prior knowledge is of paramount importance to rapidly develop new vaccines as it provides a platform-based framework beyond product-specific information [11,41]. In a pandemic scenario, the use of consolidated platforms for the rapid execution of developability assessments, formulation studies, and process development is essential. These workflows assess the antigen liabilities and identify the simplest composition to stabilize vaccines and improve their delivery and manufacturing. An internal database can be built, containing all compositions and process parameters of lyophilized vaccines with a focus on recombinant proteins and glycoconjugate-based vaccines (commercial and vaccines under development). This database enables the identification of a standard vaccine composition by using standard protocols and thus standard parameters for lyophilization that can be rapidly adapted to new vaccine candidates. Furthermore, the feasibility of a freeze-dried formulation is assessed in the early stage, recognizing the intrinsic diverse nature of protein antigens.

In the early phase of vaccine development, minimal information on the new vaccine candidate and its stability is available. However, due to the favorable stability of lyophilized products compared to liquid formulations, a parallel path for liquid and lyophilized presentations during early development might be considered. In general, a liquid formulation in a vial or prefilled syringe presentation is typically preferred for commercialization and ease of use, thus feasibility assessments for such presentations during early development could benefit acceleration. In the late stages of development, it is important to understand the process parameters and their impact on the CQA within the expected variability. As manufacturing several doses in a short time frame is expected during a pandemic, there is a need to expand the manufacturing network to cover the demand. As the number of manufacturing sites in the network grows, particularly in a pandemic scenario, a detailed understanding of the process is required to document the comparability of the batches and validate the process for vaccine commercialization. Finally, we deem that the upfront selection of a proper container closure system (e.g., a vial/stopper combination), which has already been assessed in terms of machinability and compatibility with the formulation, will further expedite vaccine development.

#### 2.2.3. Analytical Platforms

The paradigm learned by the last pandemic on the criticality of rapid access to new vaccines and pharmaceuticals is true also for the development of analytical tools: rapid delivery of new products is based on rapid development, and here, the use of prior knowledge plays a key role in increasing efficiency and allowing smart risk choices [11].

Analytical platformization is a relevant tool in structuring (analytical) prior knowledge to support an accelerated development timeline with reduced resources, in a functional, accessible way, self-adapting through the continuum feedback of life-cycle monitoring.

**Figure 3:**
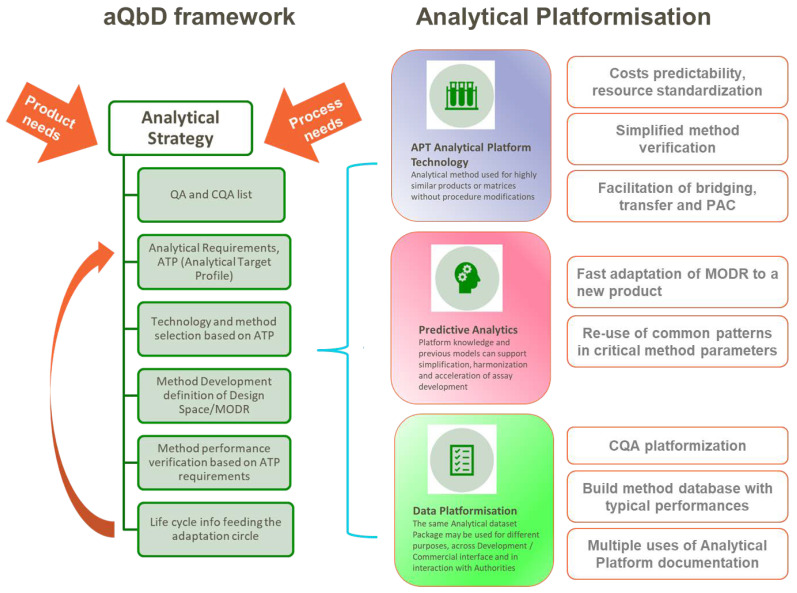
Graphical summary of analytical platformization opportunities in the context of analytical QbD framework, based on the cited literature.

Injected into the systematic approach to build in quality from the start provided by the application of QbD principles [13], analytical platformization works as an accelerator for several elements, as summarized in Figure 3:

The analytical QbD framework allows for decision-making using a science- and risk-based approach, helping to deliver a robust and fit-for-purpose analytical procedure across the life cycle of its use. [42,43,44]. The cornerstone of aQbD (analytical quality by design) is the pre-definition of the method requirements via the ATP (analytical target profile) against the identified product QA/CQA (quality attributes, critical quality attributes). The ATP is informed by product/process requirements (e.g., specification acceptance criterion for a quality attribute) and is not linked to any specific analytical technique. The selection process will be then driven by prior knowledge and/or experimental verification relating to the performance of available analytical technologies. Once the technique is selected, procedure-specific performance indicators can be defined that will support method development and ensure the performance criteria in the ATP are achieved [42,43]. As a final step, life-cycle information from the method used may give feedback on the cyclic optimization of analytical conditions.

The most obvious aspect of analytical platformization is the use of analytical platform procedures: “A platform analytical procedure can be defined as a multi-product method suitable to test quality attributes of different products without significant change to its operational conditions, system suitability, and reporting structure”, as reported n the ICH Q2(R2) Step 2 draft [45].

Benefits to exploiting the similar properties of different candidate vaccines and building platform methods around relevant standards, whereby the hardware, consumables/reagents, software, and the underlying methods are all standardized, will provide drug manufacturers with numerous benefits:

- Shorter time to market (faster development)
- Higher cost predictability for each new product
- The ability to standardize operations and staff training
- Simplified and reduced method performance assessment
- Less bridging and characterization activities, easier comparison between different products
- Easier and faster method sharing across different sites
- Less waste

One aspect particularly cumbersome when trying to accelerate development is the need to demonstrate an analytical method fit for purpose through a method performance assessment exercise (qualification, validation) for any new product; the use of analytical platforms may relieve and speed up the process in several ways [13,42,43,46]:

- Design the analytical method as a platform from the start to cover multiple products, for example by the use of representative commercial standards to demonstrate performance (when possible, e.g., for assessing the dimensional range for analytical sizing) instead of specific internal standards.
- Use risk-based evaluation for the potential impact of new product(s) characteristics on qualification/validation parameters (e.g., no changes in the matrix for new product = no changes to specificity).
- Apply prior knowledge: method suitability confirmation can be sustained with prior knowledge derived from past platform method applications, without the need for an experimental repetition (if justified e.g., by risk assessment).
- Produce agile (modular) documentation: keep the original qualification/validation report, adding a “new module” containing the relevant information for any new product (e.g., as an appendix to the original document) without the need to replicate it completely.

This framework can be considered for any vaccine type and test attribute, with product-specific considerations evaluated in the initial risk assessment mentioned above. For example, for an mRNA platform, a chromatographic or electrophoretic separation procedure for purity assessment could have the same critical method parameters [13] but tailored operating conditions may need to be developed, depending on the specific product. On the other hand, if the output of the risk assessment does not require a full re-development of the analytical procedure (e.g., some well-known safety-related attributes), platform knowledge can be readily applied [47].

Another area of application of platform knowledge for simplification, harmonization, and acceleration of assay development is the use of predictive analytics: the output of successful method development is the identification of MODR (method operable design region) [13,43], the operating range for the critical input variables that produce results consistently meeting the ATP requirements, ensures optimal method performance and identifies a region around the selected method conditions, which is likely to be robust over its life cycle.

Defining MODR for an assay requires the identification of the critical method parameters (CMPs) through modelling studies. The development of subsequent analytical methods based on the same technical principle (e.g., ELISA and HPLC) may generate technical platform knowledge by the identification of commonalities among critical method parameters, and the re-use of these generic patterns can be injected into future method development across multiple assets to simplify and accelerate delivery of fit for purpose assays [13].

Moreover, solid knowledge of MODR permits the flexibility to provide the expected method performance in a modified environment. For example, evaluating the effects of variation of a potential critical method parameter within the boundaries of an established MODR allows for the quick optimization of the new required conditions to satisfy ATP while maintaining the analytical knowledge previously collected and can eventually support an abbreviated method verification exercise [13,43,46].

Structuring of prior knowledge works very effectively in the area of (analytical) data platformization. For example, a standardized list of CQAs for products belonging to the same molecular family may be used as a starting point to speed up the definition of the final (analytical) control strategy for a new similar vaccine antigen; or an up-to-date database of analytical methods and their typical performances used to test a specific quality attribute can facilitate method screening for a new candidate; as mentioned above, related to platform validation reports, a modular analytical dataset package may be used for different purposes, across the development/commercial interface and even in the interaction with authorities.

All these aspects also show a good fit with the evolving scientific and regulatory trend towards a stronger, scientifically based risk-management analytical culture where the use of (structured) prior knowledge assumes more and more relevance [44,48].

### 2.3. Best Use of Innovation and Digital Solutions

This section will focus on specific innovations and CMC strategies that are deemed relevant for vaccine acceleration, based on a literature assessment and the authors’ experiences. Some of these examples refer to the two vaccine platforms described above (e.g., novel cell line development for CHO platform, formulation prediction for subunit protein vaccines, and mRNA optimization), while others could be broadly applicable to any vaccine type (e.g., stability modeling and digital twins).

#### 2.3.1. Use of AI, Data-Driven Computational Modeling, and Digital Platforms

A particularly promising way to leverage prior knowledge is by developing machine learning (ML) and other data-driven models. These solutions can help reduce the need for wet-lab experiments, predict experimental results, provide data-based insights, support and accelerate data-driven decision-making, and ultimately, speed up development timelines. Today, process development is still largely driven by the design of experiments and the intuition of the individuals involved. Such efforts may quickly become costly and time-consuming given the complexity of manufacturing processes and the sheer number of experimental variables to consider.

A shift in the direction of fully data-driven vaccine development is made possible by the ever-growing amount of data generated by automated equipment, sensors, and processes as well as advances in high-throughput experimentation and expanding networks of external partners and data providers. The increasing digitalization of data workflows and investments towards the continuous flow of standardized data throughout the R&D life cycle of different assets from early research to manufacturing promises to enable data-driven investigations into how upstream process parameters affect downstream manufacturing and even patient outcomes [49].

One approach to realizing this potential involves building “digital platforms” that can reuse data produced across assets, both internally and externally, and impact all or almost all portfolio assets within the same modality or another well-defined applicability domain. This approach efficiently leverages prior knowledge and can be particularly useful in early development where traditional methods face numerous challenges due to limited knowledge of a given molecule or antigen as well as limited access to experimental material.

To illustrate the points made above, we can consider the case of mRNA vaccine optimization at the drug substance level [50]. Accumulating evidence shows that some of the remaining key challenges of the mRNA technology (including poor thermostability, reactogenicity, and limited protein production), are intrinsically linked to the properties of the mRNA molecule itself. In particular, these key vaccine properties are largely affected by the RNA sequence and structure, both of which can be manipulated and optimized [2,32,51,52]. Optimizing mRNA sequences for vaccine design presents difficult combinatorial challenges due to the extremely large selection space. Specifically, for the antigen-coding sequences, there can be exponentially many mRNA sequences that encode the same protein due to the degeneracy of the genetic code and the independent codon choice for each amino acid. For instance, there are more than 10^632^ possible mRNA sequences that produce the spike protein of coronavirus, the target antigen of the COVID-19 vaccines—a much larger number than the estimated number of atoms in the visible universe [53]. This large space of possible solutions could not possibly be explored using traditional experimental methods, but computational models can efficiently sample through this space in search of the best vaccine candidates.

Machine learning and other types of computational methods can be built to identify sequence and structural patterns associated with important mRNA properties based on experimental data from various mRNA molecules, including vaccine candidates, mRNAs encoding model proteins, and even natural human transcriptomes. In addition to offering novel insights into RNA biochemistry and molecular biology, computational methods can also be developed to optimize mRNA molecules to improve their relevant properties. mRNA vaccine sequences optimized using such methods are significantly different from the original sequences derived from the pathogen’s genome, whilst still encoding for the same pathogen protein and offering good immune protection. Compared to the wild-type sequence, the optimized mRNAs may demonstrate, for example, increased codon optimality, leading to increased protein expression in human cells, or higher levels of secondary structure, linked to improved stability in solution [32,37]. Both mRNA vaccines approved in the early days of the COVID pandemic have been computationally optimized, which led not only to improved product characteristics but also shorter development timelines [54]. For example, Moderna’s previously developed sequence optimization platform technology enabled the mRNA sequence of the Spikevax vaccine to be designed in just one hour. The company was confident in its digital platform methodology and progressed from a single candidate to manufacturing whilst conducting in vivo studies in parallel [55].

The proposed approach could represent a key asset also for the rapid response to new virus variants, allowing for a fast and optimized mRNA design. There are numerous other examples where artificial intelligence and machine learning have started bringing value to the technical development of therapeutics: from using a similar sequence modeling strategy to increase the yield of protein vaccine production in cell cultures [56] to the automated inspection of product defects based on analysis of their automatically generated images with computer-vision technology [57].

To fully exploit the potential of machine learning in CMC, the key is to invest in improved data strategy and infrastructure. Currently, despite the production of large amounts of data in screens and processes across technical development, the generated data are still typically only used to address specific inquiries and are seldom reused to bring value to future assets or related applications. This problem can largely be attributed to legacy processes, siloed data and systems, and lack of data standards, all of which make the data difficult to find, access, and reuse by both people and algorithms.

To overcome these challenges, companies need to invest in physical automation and new equipment, such as liquid handling stations, progressive digitalization, implementation of robust fit-for-purpose data governance and knowledge management frameworks, and capabilities to train machine learning models with limited CMC data, including active and transfer learning. Other areas of improvement include the development of a culture of effective data sharing between departments, safe precompetitive data sharing, for example, based on the technology of federated learning, and, crucially, the ability to attract, nurture, and retain talent in the highly competitive area of data science.

In summary, CMC organizations must recognize data as a strategic asset and manage it accordingly. This shift will facilitate the development of next-generation digital platforms and enable the integration of data science at the core of vaccine development.

#### 2.3.2. Stability Modeling

The stability evaluation of pharmaceutical products is a key quality requirement to ensure the efficacy and safety of the products during their intended period of usage and under the recommended storage conditions.

Guidelines on stability are notably proposed through ICH Q1A-Q1F documents, ICH Q5C for biological products, and WHO/BS/06.2049 for vaccines. Those documents give guidance regarding the stability studies and associated data packages to establish the adequate storage conditions (e.g., temperature) and the shelf life for the product; the shelf life is defined in WHO/BS/06.2049 [58] as “the period of time during which a vaccine if stored correctly, is expected to comply with the specification as determined by stability studies on a number of batches of the product”.

For vaccines, refrigerated storage (e.g., 5 °C for the final drug product) is commonly applied to remain stable. From WHO/BS/06.2049, the stability evaluation is performed through a real-time, real-storage-condition study that should cover a minimum period of 6 months at submission. A minimum of 12 months is even mentioned for the case of release specification modeling.

Therefore, in a pandemic situation, stability assessment based only on real-time studies strictly following the current regulatory framework may likely be on the critical path for accelerated development of vaccines and for rapid access to patients.

A solution to overcome this bottleneck could be a more systematic application of accelerated stability studies (i.e., from WHO/BS/06.2049, “studies designed to determinate the rate of change of vaccine properties over time as a consequence of the exposure to temperatures higher than those recommended for storage”) performed over a shorter period (e.g., not more than 6 months) and completed by advanced kinetic modeling and statistical approaches.

Indeed, such advanced modeling of stability data generated at different accelerated conditions (i.e., at higher temperatures than 5 °C such as 25 °C, 37 °C, 45 °C) and at different time points over a short period establishes the evolution of the attribute of interest whatever the temperature and its exposure period in a single model. Hence, it can predict the quality of the product at the end of its shelf life at long-term conditions (i.e., at 5 °C). Such an approach would avoid the completion of long-term stability studies to establish shelf life, supporting acceleration in vaccine development and supply [14]. This is obviously under the assumption that the higher selected temperatures, which accelerate the degradation, are representative (i.e., they do not generate any additional degradation pathway compared to the long-term condition), thus allowing a relevant prediction at the long-term condition.

As an advanced kinetic modeling approach, the Šesták–Berggren model based on a differential equation can be applied [59]. It can be expressed as:

$$\frac{d\alpha }{dt}=Ae^{\frac{-E}{RT}}{\left(1-\alpha \right)}^{n}$$

where $\frac{d\alpha }{dt}$ is the degradation rate, α is the reaction progress, *A* is the pre-exponential factor, *E* is the activation energy, R is the universal gas constant, and T the temperature (in Kelvin).

**Figure 4:**
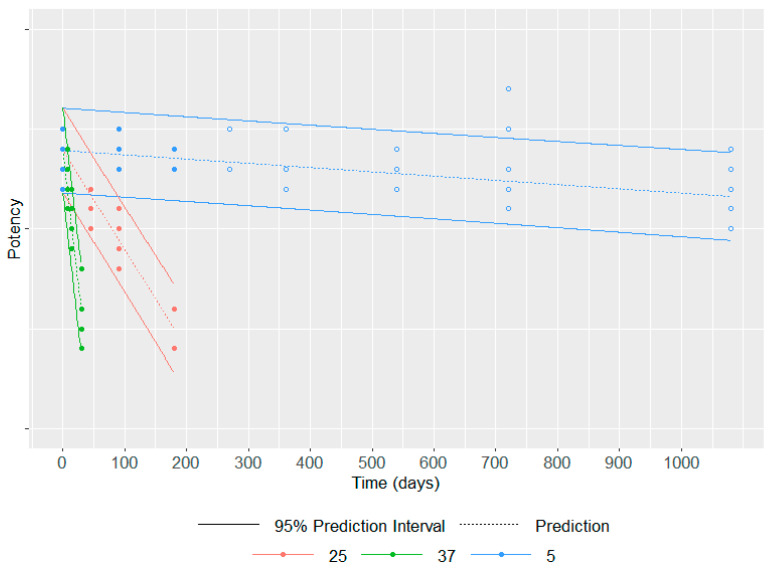
Stability data at 5 °C, 25 °C and 37 °C up to 6 months (filled circles) were used to model the potency loss for each temperature over time represented by the dashed line and its 95% prediction interval represented by solid lines; the data at 5 °C after 6 months (empty circles) are used to assess the quality of the model.

As an illustrative example (Figure 4), this model has been applied to six batches of a commercial vaccine. An accelerated stability study has been performed over 6 months (precisely 193 days) at 25 °C and 37 °C, in addition to the classical long-term study at 5 °C up to 3 years. The Šesták–Berggren model has been applied to all stability data of potency up to 6 months regardless of the temperature, i.e., 5 °C, 25 °C and 37 °C (data represented by filled circles). Of note, data from all batches have been pooled to build this model as no different behavior over time is expected among batches for each temperature. For a given temperature, the dashed line represents the potency predicted by the model at a given time point, and the solid lines represent the 95% prediction interval. The potency data at 5 °C after 6 months i.e., data not considered in the model establishment (data represented by empty circles) are for most of them within the 95% prediction interval. This example highlights that the potency loss over time can be adequately modeled for up to 3 years based on only 6 months of accelerated and long-term data.

This example, among others [14,59], demonstrates how advanced stability modeling combined with statistical approaches to consider model uncertainties can be of precious help to predict the stability of a product at long-term conditions based on data generated over a restricted period. Therefore, this approach becomes a critical asset to support the acceleration of vaccine development and supply, especially in the case of pandemic emergencies and unmet medical needs.

Note that in addition to the extrapolation of shelf life for new vaccines, this modeling approach can be applied to other objectives. It can be used to establish release specifications, to characterize product understanding of the degradation pathways, and to support the management of post-approval changes. Additionally, as a single model is built establishing a relationship linking time and temperature to the quality attribute of interest, the impact on the product exposed at any temperature (i.e., other than the temperatures used to build the model) during a given period can be easily estimated. Therefore, such a model can also be used to support supply chain management with respect to the monitoring of the quality of vaccines during shipping, especially by estimating the impact of potential cold chain breaks.

To make the application of accelerated stability modeling successful, the establishment of the experimental plan is of critical importance: the accelerated temperatures should be adequately selected allowing to exhibit a degradation over a short period while remaining representative of such degradation at long-term conditions; the frequency of time points as well as the number of analytical replicates at each condition should be established based on prior knowledge (regarding, e.g., preliminary information about the degradation pathway and its kinetic particularly in the context of a vaccine platform, estimation of the assay, and batch to batch variability) and based on a statistically powered analysis performed in line with the pre-established objectives of the study.

To conclude, the application of advanced accelerated stability modeling is a valuable and reliable approach to predicting stability and a key approach to accelerating vaccine development. However, regarding the regulatory framework, while the use of accelerated studies is mentioned in ICH Q5C [60] (“the expiration dating should be based on real-time/real-temperature data. However, studies under accelerated conditions may provide useful support data for establishing the expiration date”), there is no further guidance to design those studies, and no clear incentive to apply advanced kinetic modeling on those accelerated stability data (e.g., from WHO/BS/06.2049, “accelerated degradation testing should be seen as a support to real-time conditions studies and not as their replacement”).

Based on an increasing number of successful applications of stability modeling, the ongoing discussions between industry and some regulatory agencies should continue to widen its use through an updated regulatory framework. Such advanced stability modeling could be thus systematically applied to accelerate vaccine development and supply.

#### 2.3.3. Novel Cell Line Development Workflow and Cell Expression Technologies for CHO Platform

##### Novel Cell Line Development Workflow

**Figure 5:**
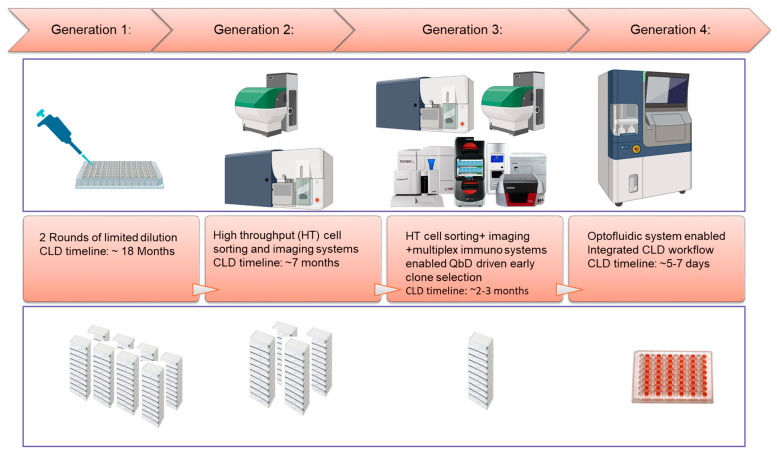
Evolution of cell line development (CLD) workflow for CHO protein vaccines.

ICH recommends the recombinant-protein-based drug substance for clinical trials and beyond. Recombinant proteins are produced from a clonally derived stable cell line to ensure the consistency of the stable product quality attributes [61]. To achieve this goal, a rigid cell line development (CLD) workflow with single clone derivation and clone selection process are typically performed, involving significant manual operations, such as rounds of limited dilutions for single-cell derivation followed by plate and shake-flask-based batch culture formats for selecting clones with desirable productivity and quality. This conventional approach has low throughput, is labor intensive, and is time-consuming. Over the past few years, major technology advancements have been made to streamline and accelerate cell line development workflow for vaccine development. Two examples are highlighted below: (1) The utilization of multiple high-throughput analytics (combining a fluorescence-activated single-cell isolation approach and a multiplex immuno-tool) and cell imaging systems (clonality evidence generation) to enable early assessment of cell line process and product quality attributes, therefore, expediting lead clone selection [62]. This approach has been quite robust, though it still requires multiple standalone instruments and a significant amount of manual operation, including manual maintenance and scaling-up of hundreds of clones at the mini-plate stage. (2) More recently, an emerging cell line development technology utilizing the optofluidic system (such as Beacon technology) has demonstrated its feasibility to enable rapid generation of commercial cell lines for vaccine production with a highly automated and integrated workflow [63] (Figure 5).

##### Expression Systems

**Figure 6:**
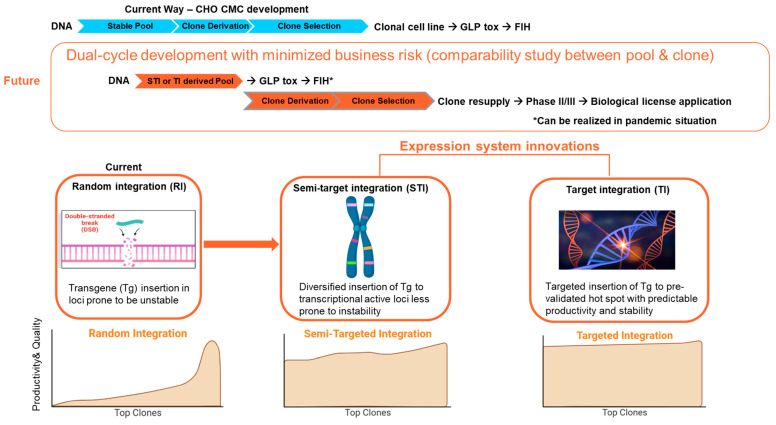
Innovations in expression systems enable dual-cycle development strategies. GLP Tox: Good-Laboratory-Practices toxicity batch. FIH: First in human clinical trial.

Recent advancements in cell line expression technology, such as target-integration (TI) and transposon-based semi-target integration (STI) platforms, have demonstrated the capability to produce cell substrates with highly homogeneous and predictable performance. Comparability between the stable pool and derivative clones has also enabled a dual-cycle development strategy that may become the new “norm” for CMC development: using the non-clonal stable pool to initiate the drug substance production for preclinical study (toxicity study) and even early clinical trials (Phase I); whereas clones were resupplied for late-stage clinical trials and commercialization [64,65,66,67,68,69] (Figure 6). It is conceivable that the implementation of this dual-cycle development strategy could enable a 2–3 month “timeline saving” for the clinical entry of vaccine candidates.

#### 2.3.4. Modeling to Support Formulation, Process Development, and Innovative Control Strategies

##### Formulation Modeling to Develop Formulation Composition Faster

Formulation development usually includes multiple steps, from the new antigen’s first solubility evaluation in different buffers (discovery phase) to the final compatibility with the final container (development phases for clinical trials). Most of the formulation composition determination work is based on empiric evaluation through the HTP (high throughput) screening process, on skilled scientific experience, and on excipients that are usually already available in defined raw material stocks. The formulations are also stressed versus manufacturing conditions, such as shear induced by agitation, oxidation due to light exposure, residual peroxides in formulation, or filling isolators among others. This is to anticipate and prevent manufacturing issues. All this work is time-consuming and in a pandemic situation could be on the critical path, pushing the vaccine manufacturers to initially develop freeze-dried presentations and work on a second generation of the vaccine for a liquid presentation, leading to higher costs.

Recently, with the combination of HPC (high-performance computing) and protein dynamic modeling, it is possible to simulate interactions with buffers and predict formulation stability. However, due to the high calculation needs of full atomistic models, which could take months of simulation work, a coarse-grained level has been introduced to facilitate the simulation and bring the simulation time within a few days [70,71,72]. The coarse-grained level of the 3D structure of the protein is an approximation and thus it loses some degree of information related to the atoms’ positions, which are replaced by amino acid positions or beads containing several amino acids. However, for interaction studies, this approximation offers sufficient results.

A mechanistic model of the protein’s aggregation can be defined within a range of pH and buffer compositions, including salts and other excipients. Dynamic protein simulation software can be used for building such models with the introduction of temperature stress tests in order to have a prediction of the long-term stability of the protein. When analyzing the aggregation interface, the nature of the aggregation can be defined, helping in such a way that ad hoc excipients can be proposed to the scientist to avoid aggregation, such as surfactant or sugars.

The mechanistic model is then validated by experimental evaluation. Predicted formulations are prepared to confirm the aggregation levels and the efficacy of identified excipients to prevent such aggregation if needed. Once it is validated, the model can be used to produce a lot of in silico data that will be used to teach a machine-learning (ML) model.

One needs to consider the limitations of the mechanistic models in terms of what can be simulated. It cannot yet predict what would happen in case of exposure to oxidizing conditions (e.g., residual peroxides in filling isolators) or shear exposure due to agitation of the formulation in manufacturing vessels. Therefore, it is important to complement the mechanistic model with an empirical model, testing in vitro other stress tests for the candidate formulations.

The two models can be used to generate data that will be used to train the ML model, which can be used later to predict the best formulation in terms of stability, giving more chances to initially develop liquid formulations to decrease the overall costs and time to market.

Developing models for the formulation behavior, containing antigens but also adjuvants, will increase product knowledge and associated quality as well as reduce write-offs due to deviations.

##### Continuous Formulation Development and the Digital Twin Use to Connect to the Filling Line

Continuous manufacturing (CM) is gaining increased attention for the production of pharmaceutical and biologicals, including vaccines, at the drug substance and drug product level. CM development is supported by regulatory authorities, see recent ICH guidance Q13 [73]. CM holds the promise of multiple advantages, such as a small footprint, more sustainable due to lower water and energy consumption as well as lower global warming potential. Additionally, CM brings higher opportunities for digitalization through modeling and PAT introduction, and in the context of the pandemic, significantly decreases the time to market. Indeed, considering the significant redevelopment work must be carried out when changing the scale of the process, having a single scale from R&D to the final manufacturing site speeds up the transfer of the process significantly.

PAT is critical to enable CM as described for different examples of solid dosage manufacturing [74] or flow-chemistry [75] where the selection of the PAT is based on the analyzed CQA and potential risks impacting the CQA.

**Figure 7:**
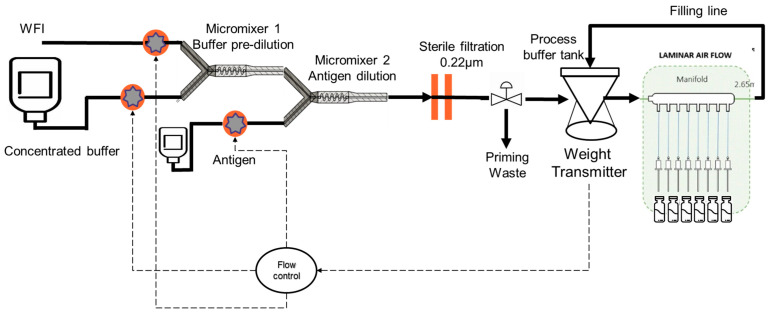
Continuous formulation connected to a filling line.

The continuous formulation of vaccines is possible using accurate dosing pumps in combination with static mixers. The right proportions of each excipient, antigen, and adjuvant are defined by the flow rates of the pumps, which are considered critical process parameters (CPP). As these CPPs impact the final vaccine composition (CQA), flow-rate meters were introduced into the formulation assembly. Homogeneity is ensured by the mixing efficiency of the in-line mixers. The system can be further improved by connecting the formulation assembly to a filling line so that no final bulk is accumulated and it is directly filled into vials or syringes (see Figure 7) via a buffer bag or small tank.

However, there are multi stops and starts at the filling line due to various technical issues that may arise during the filling while the formulation should remain continuous to avoid the loss of product each time the process stops and restarts. To solve this issue, the total flow rate of the formulation part can be adapted to the need of the filling line (defined by the buffer bag weight evolution) by decreasing or increasing and using the buffer bag to cover some duration of the filling stop.

In that specific setup, the control of the transitions of the flow rate is critical to keep the composition of the formulation within specification. To achieve that, a digital twin can be developed to master the process and the transitions and predict all relevant critical quality attributes (CQA) and potential failures.

The digital twin is the digital equivalent of a process, receiving data from the process, giving insights, and sending actions to the real process. It can include computational fluid dynamic (CFD) simulations and mechanistic models, including dynamics of the pumps, which can be validated with experimental results. In silico and experimental data can be used to train ML models that can be used as part of the control strategy of the continuous formulation connected to the filling process. An interesting review was made by Yingjie Chen et al. [76] for the application of digital twins in pharmaceutical and biopharmaceutical manufacturing where the selection of PAT and process modeling are discussed.

The digital twin is also based on inputs coming from process analytical technology (PAT) sensors which can be in/on-line or at the line. Conductivity sensors can be used to monitor the right dilution factor of the buffer or antigens. A UV detector can be used on-line and measure the concentration of a protein with the support of a chemometric model.

However, there are still some regulatory challenges to face before this kind of process can be introduced in GMP vaccine manufacturing, such as lot definition, diversion strategy in case of out-of-specification detection, the geometry of the equipment, the resident time distribution of the product, the time-to-results speed of the PAT sensor, and the reaction time of the three-way valve. Other challenges include the introduction of process models, including digital twins, into the registration file and how models can be updated after data accumulation at the manufacturing site.

The concept of continuous manufacturing can be extended to the mRNA platform in the context of the pandemic. The mRNA must be protected from degradation and delivered to the cytosol of target cells where it can be translated into the antigen-protein. This is best achieved through encapsulation into an LNP (lipid nano particle) that is specifically designed through the choice of lipids that captured the mRNA inside the lipidic vesicle but also drives the endosomal escape to the cytosol to reach the ribosomes.

However, if one looks at the different process steps for the manufacturing of mRNA-LNP vaccines, the lipid nanoparticle formation process itself is by nature continuous. Ethanol-containing lipids are continuously mixed with an aqueous buffer containing mRNA to be encapsulated in microfluidic devices, T-junction, or other mixing devices. The lipids precipitate entrapping, through the presence of ionizable lipid, the mRNA. The mixing quality directly modulates the size distribution and the encapsulation yield, impacting the stability and the potency of the vaccine.

The continuous LNP generation process can further be connected to the continuous formulation and filling process. One example of such a process is discussed by Aline Hengelbrock [77] where different methods of LNP generation and ethanol removal are presented.

To obtain a fully continuous end-to-end (E2E) process, the synthesis of the mRNA must also be included in a continuous manner. The DNA template could be considered the starting point of entry for the mRNA process (see Section 2.2.1 for the batch process). The desired sequence coding for the desired protein to be expressed has been inserted into the DNA, which could be in the form of a plasmid if the amplification process is based on *E. coli* fermentation or linear if PCR (polymerase chain reaction) techniques are used. For continuous processes, enzymatic methods look more promising as they do not depend on the fermentation of producing bacteria and an extraction followed by a purification process, which is more appropriate for batch production. The DNA is then used as a template to produce the mRNA in an enzymatic reaction. Once the mRNA is produced, it must be purified to remove mRNA fragments or double strands of mRNA that may induce reactogenicity upon injection. The purification method can be based on a multi-column setup to allow continuous multiple injections of the product combined with an oligo-dT chromatography column [78]. DNA immobilization on a support could lead to a lower need for DNA amplification as it could be used for a high number of transcription cycles easing the transformation of the batch process to a continuous form. Nonetheless, the complexity of the automation part must not be underestimated as well as the connections between the different steps.

The continuously automated mRNA LNP manufacturing process could positively impact the cost, mainly by personnel and consumable reduction [79]. The combination of continuous mRNA, LNP generation, formulation, and filling operations could lead to a globally and significantly smaller footprint of mini-factories, which could be rapidly deployed over the globe wherever the potential next pandemic could strike.

## 3. Discussion and Concluding Remarks

This review has provided an overview of CMC strategies and technical enablers to support accelerated access to vaccines. The successful implementation of these approaches depends on the ability to integrate the different aspects holistically, with cross-disciplinary synergies clearly identified, and an agile prioritization framework and governance to make fast decisions without compromising safety and efficacy. In addition, these elements need to be combined with improved and streamlining of operational processes, and the extensive use of robotics and automation technologies.

Sustained acceleration and pandemic preparedness require broad acceptance of the proposed strategies by regulatory agencies worldwide. This can be achieved through continued dialogue across the industry to establish common positions on innovation and CMC strategies, supporting the establishment of new standards and coherent messaging to regulators. Finally, it will be key to build on the existing opportunities and learnings offered by some regulatory agencies to support accelerated development and foster reliance and harmonization across different regions.

Beyond the technical innovation that supports acceleration, it will also be important to adapt the industry model of development, considering, for instance, a holistic end-to-end program strategy, the commercial strategic intent from the start, the minimum valuable product, the process for each development stage, and a plan to assess the highest probability of success [80].

## Data Availability

No new data were created for this work, due to the nature of the work (review).
